# How plants pick their friends

**DOI:** 10.7554/eLife.108116

**Published:** 2025-07-17

**Authors:** Estelle Grundy, Michael Udvardi

**Affiliations:** 1 Queensland Alliance for Agriculture and Food Innovation, University of Queensland St Lucia Australia

**Keywords:** legumes, nitrogen fixation, rhizobia, symbiosis, plant immunity, RIN4, Other

## Abstract

A protein called RIN4 has a central role in helping legumes such as soybean and the bacteria rhizobia to develop a mutually beneficial relationship.

**Related research article** Tóth K, Kim D, Cho S-H, Nguyen CT, Nguyen THN, Hartanto C, Michno J-M, Stec AO, Stupar RM, Stacey G. 2025. Soybean RIN4 represents a mechanistic link between plant immune and symbiotic signaling. *eLife* **12**:RP93149. doi: 10.7554/eLife.93149.

Plants rely on specific microbes, such as bacteria and fungi, to capture nutrients from their environment (Udvardi and Poole, 2013; Harrison, 1999). These microbes are accommodated wholly or partly within cells of the plant, which is noteworthy because plants have elaborate defence systems to protect themselves against bacteria, fungi and other pathogens. Intriguingly, even beneficial microbes like rhizobia – the bacteria that help plants fix nitrogen from the atmosphere – trigger these defences. However, rhizobia quickly suppress this response, allowing it and the plant to live a symbiotic relationship, with each supplying the other with nutrients they need to survive (Libault et al., 2010).

Previous work has shown that plants recognize beneficial microbes via cell surface receptors that bind molecules specific to each microbe, but the processes by which these microbes go on to bypass the immune defences of the plant remain poorly understood (Zipfel and Oldroyd, 2017; Bozsoki et al., 2017). It is known that plants in the legume family, such as soybean, detect rhizobia via molecules called Nodulation (Nod) Factors. Detection triggers a signalling cascade in which several proteins are sequentially phosphorylated, resulting in the activation of the genes required to establish a symbiotic relationship. Establishing this relationship involves the development of specialized plant organs called root nodules that accommodate and feed the rhizobia. Now, in eLife, Katalin Tóth, Gary Stacey and co-workers at the University of Missouri and other institutes in the US and Vietnam report that a key plant immunity regulator, RIN4, is phosphorylated soon after contact with rhizobia to promote symbiosis (Tóth et al., 2025).

RIN4 has a dual role in a process called PAMP-triggered immunity (where PAMP is short for pathogen-associated molecular patterns). Under normal conditions, RIN4 suppresses PAMP-triggered immunity, presumably to avoid the cost of unnecessarily activating the plant’s defences (Ray et al., 2019). But when phosphorylated by PAMP receptors, RIN4 switches roles and activates the immune response instead.

To investigate if RIN4 is also involved in symbiosis, Tóth et al. used two genetic tools, CRISPR-Cas9 and RNA interference, to either eliminate or reduce RIN4 activity in soybean plants. They found that this reduced the expression of transcription factors required for nodulation, resulting in fewer root nodules being produced. Additionally, overexpressing RIN4 increased nodule formation.

Further experiments revealed that phosphorylation of RIN4 is critical for its role in nodulation. When rhizobia come in to contact with a root cell, RIN4 becomes phosphorylated at a specific site – a serine amino acid at position 143 (S143) – that is only found in legumes and other nitrogen-fixing plants. Tóth et al. found that soybean plants with a mutated form of RIN4 that cannot be phosphorylated at S143 developed fewer root nodules compared to wild-type plants.

Importantly, this phosphorylation event is carried out by an enzyme called SymRK (short for symbiosis receptor-like kinase), which is a key component of the receptor complex that detects the Nod factors produced and released by rhizobia. As well as helping legumes form root nodules, SymRK supports symbiosis between plants and certain fungi**,** and has also been shown to suppress immunity in the legume *Lotus japonicus* (Feng et al., 2021). On top of this, a recent study of the legume *Medicago truncatula* discovered another component of the Nod factor receptor that, along with a receptor kinase, suppresses immune signalling (Wang et al., 2025). Taken together, these studies highlight the diverse ways in which beneficial microbes can subvert the defence systems of plants, and the important role that Nod factor receptors play in this process.

Based on their findings, Tóth et al. proposed a model for how RIN4 promotes symbiosis (Figure 1). When the receptor complex recognizes Nod factors released by rhizobia, SymRK is activated, which leads to the phosphorylation of RIN4 at amino acid S143. Effector proteins from rhizobia – which are yet to be identified – then interact with this phosphorylated form of RIN4 to inhibit the plant’s defence system. This immune suppression allows rhizobia to promote the formation of root nodules and develop a symbiotic relationship with the plant. Previous studies have also suggested that microbial effector proteins allow RIN4 to regulate interactions between plants and microbes (Selote and Kachroo, 2010; Chung et al., 2014) – but these effectors, and their corresponding genes, are yet to be found.

**Figure 1.**
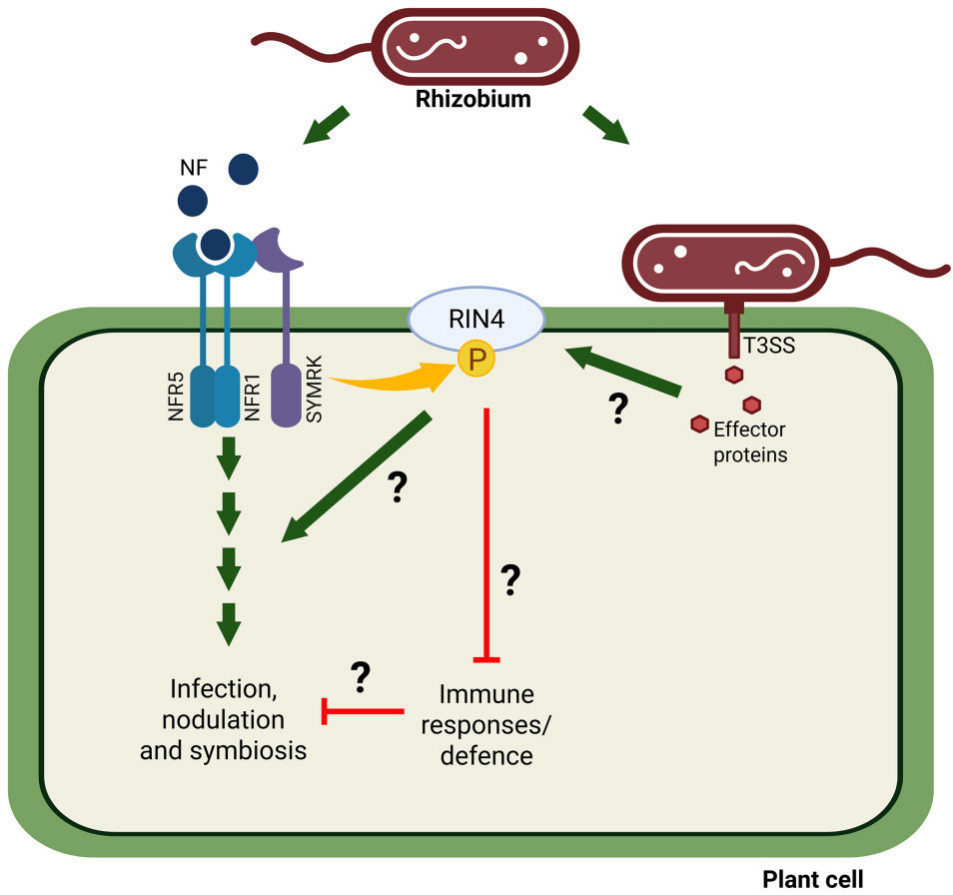
Avoiding the immune defences of a plant to establish a symbiotic relationship. Plants detect rhizobia bacteria (top) via molecules called Nodulation Factors (NF; dark blue circles), which bind to specialized NF receptors on the surface of plant cells (NFR1 and NFR5; light blue and darker blue respectively). This interaction activates the protein SymRK (purple), which then phosphorylates (yellow arrow) the molecule RIN4 (light blue oval). Phosphorylated RIN4 suppresses the immune defences of the plant (red line), allowing rhizobia to infect the plant and promote the formation of root nodules. However, it remains unknown how RIN4 represses the plant’s defences. One possibility is that effector proteins (red hexagons) secreted via the rhizobium’s type-III secretion system (T3SS) interact with RIN4 to reduce the immune response by a currently unknown mechanism. It also remains to be seen how RIN4 interacts with the symbiosis signalling pathway (small green arrows) to initiate infection and nodulation.

Created in BioRender.

An alternative possibility is that phosphorylation of RIN4 at S143 alters its structure and function, preventing it from activating PAMP-triggered immunity, without any involvement of rhizobial effector proteins. It will be interesting to see if evidence for rhizobial effector proteins that interact directly with RIN4 emerges in future studies.

The work of Tóth et al. demonstrates how legumes manipulate the protein RIN4 to suppress their defence systems when friendly microbes like rhizobia come to call. It will be interesting to see if other plant species, such as those that form beneficial relationships with mycorrhizal fungi, control RIN4 in a similar way.
